# Ultrabroadband Heterogeneous THz Quantum Cascade Laser

**DOI:** 10.1021/acsphotonics.2c01202

**Published:** 2022-12-21

**Authors:** Michael Jaidl, Maximilian Beiser, Miriam Giparakis, Martin Alexander Kainz, Dominik Theiner, Benedikt Limbacher, Marie Christine Ertl, Aaron Maxwell Andrews, Gottfried Strasser, Juraj Darmo, Karl Unterrainer

**Affiliations:** † Photonics Institute, TU Wien, Gusshausstrasse 27-29, 1040 Vienna, Austria; ‡ Center for Micro- and Nanostructures, TU Wien, Gusshausstrasse 25a, 1040 Vienna, Austria; ∥ Institute of Solid State Electronics, TU Wien, Gusshausstrasse 25a, 1040 Vienna, Austria

**Keywords:** quantum technology, optoelectronics, semiconductor nanostructures, quantum cascade laser, terahertz

## Abstract

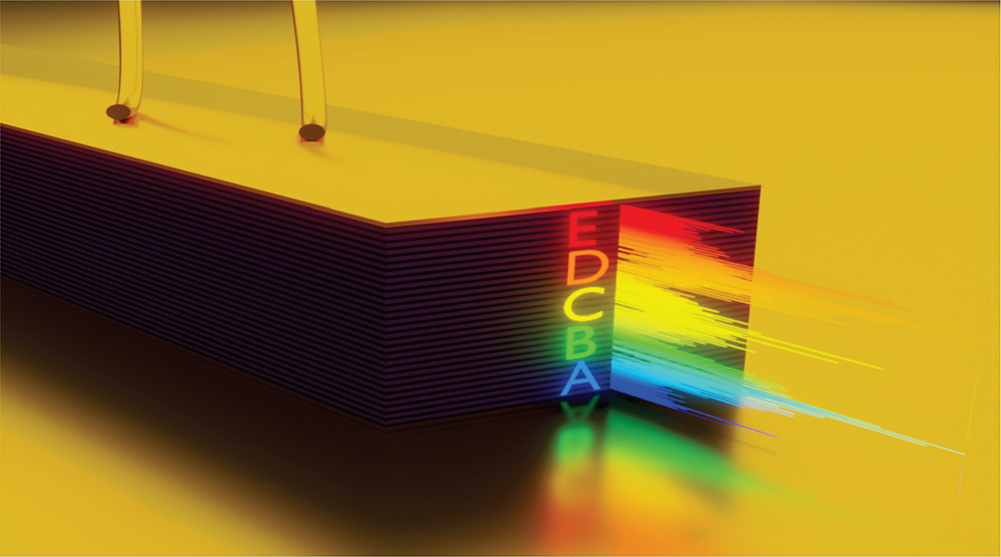

Broadband emission in the terahertz spectral region is a prerequisite for applications such as spectroscopy or white light sources. Appropriate signal powers and a compact design are advantageous for this use. A technology which meets these requirements are terahertz quantum cascade lasers. These electrically pumped, on-chip semiconductor lasers provide high output powers and the freedom of tailoring their emission wavelength by bandstructure engineering. By combining multiple active region designs emitting at different wavelengths in a single structure, one can obtain broadband emission from a single device. Here, we present a heterogeneous terahertz quantum cascade laser consisting of five individual active regions based on a three-well, LO-phonon depopulation design. The devices lase in pulsed and continuous-wave operation and emit in a spectral range from 1.9 to 4.5 THz, covering a bandwidth of 1.37 octaves. The use of the three-well design, which was optimized for high temperature operation, leads to a maximum operating temperature in the pulsed operation of 143 K.

## Introduction

The terahertz (THz) spectral region attracts increasing interest, as the development of sources and detectors in this frequency range are progressing rapidly. It is suitable for spectroscopic applications, as many molecules exhibit characteristic absorption lines in the THz band. Due to the low photon energy, THz radiation is nonionizing and therefore it is appealing for medical and security applications. THz quantum cascade lasers (THz QCLs) are reliable sources for THz emission, as they are compact, electrically driven devices and can provide high output powers >2 W.^1^ THz QCLs are made of a periodic semiconductor quantum structure consisting of materials with different conduction band offsets. These structures form quantum wells with discrete energy levels. By varying the thickness of the wells and barriers, it is possible to engineer the energy levels and to obtain a laser transition for the desired emission wavelength. The scalabity and optimization of different active region designs has been studied theoretically.^2^ An ultrabroadband THz QCL is attractive for several applications, including spectroscopy, imaging, or THz amplifiers.^3−5^ However, the active region of conventional THz QCLs consists of a periodic arrangement of several unit cells with an identical design. These unit cells can comprise two, three, four, or even more quantum wells.^6^ While these single unit cell lasers provide high gain at the designed wavelength, the emission frequency range is limited by the gain bandwidth of the chosen unit cell design. To overcome this limitation, it is possible to design active regions consisting of different unit cells designs (in the following called substacks) and stack them into a single active region, obtaining a so-called heterogeneous QCL. A crucial requirement for heterogeneous QCLs is that the currents flowing through the different substacks during lasing operation are matched. The concept of heterogeneous QCLs was introduced for the mid-infrared (MIR) range^7,8^ and later for THz QCLs. For heterogeneous QCLs in the THz region, stacked active regions with two,^9^ three,^10−13^ and four^14^ different unit cell designs have been reported. This has been demonstrated for bound-to-continuum designs. Yet a different approach has been demonstrated by using a three-well, LO-phonon depopulation design.^15^ However, this structure did not use separately designed substacks, but a chirped width of the wells by changing the GaAs growth rate for 10 unit cells, respectively. Since the threshold currents of these various unit cells were not matched due to different well thicknesses, the device did not show broadband emission; however, the emission frequency could be tuned by changing the applied bias voltage. In this Article we present an ultrabroadband heterogeneous THz QCL based on a three-well, LO-phonon depopulation design,^16^ covering the bandwidth from 1.9 to 4.5 THz. Our devices are promising for broadband coherent sources for the use in spectroscopy applications, as well as white light sources in the THz range.

## Active Region Design

The active medium consists of five different substacks of a three-well, longitudinal optical (LO)-phonon depopulation design in the GaAs/AlGaAs material system and is grown on a GaAs substrate by molecular beam epitaxy (for the growth sheet, see Supporting Information). The substacks of the heterogeneous structure are based on a structure reported in ref (16), which exhibits two intersubband transitions centered at 3.3 and 3.8 THz, respectively.^17^ This structure was optimized for high-temperature performance by adjusting the injector and the extractor level. In order to reduce electrical instabilities, the structure was designed in a way that the injector aligns before the extractor. The dynamic range was increased by 72%, and the maximum operating temperature of 196 K was reached. Based on this active region, the other four regions were designed to obtain gain at lower and higher wavelengths by adapting the thickness of the wells and the barriers. Due to the fact that our band structure calculation model does not give any information about the electric currents flowing through the simulated structure, the electrical properties of the individually designed substacks need to be investigated separately.

**Figure 1:**
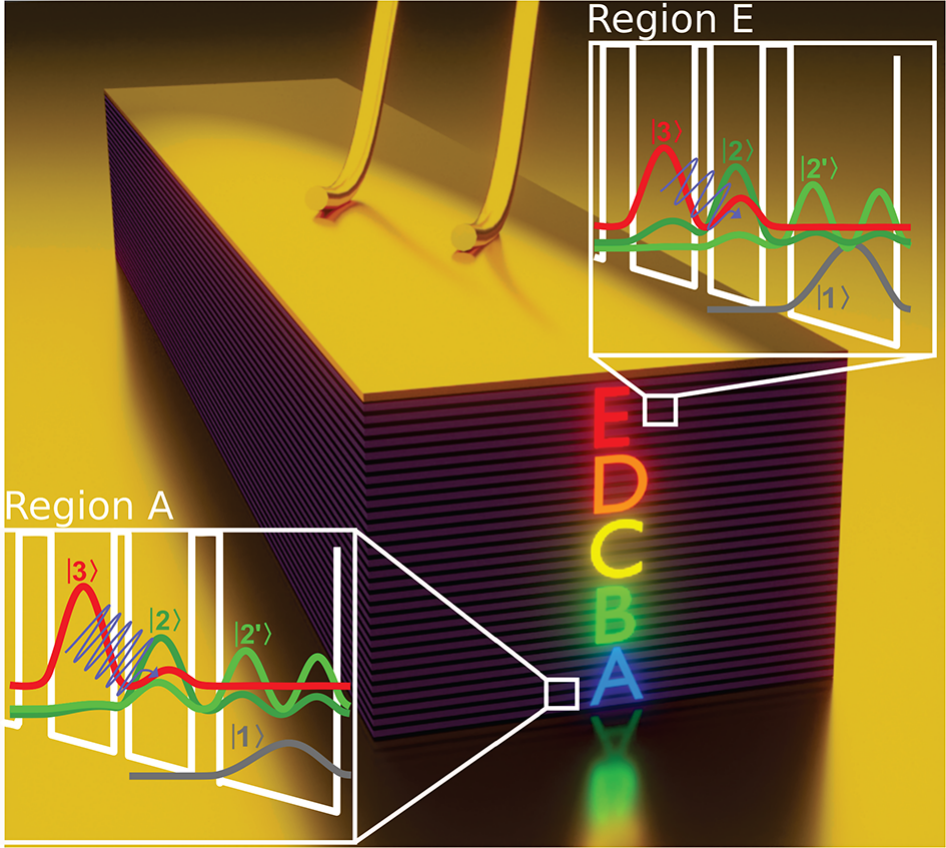
Illustration of a double-metal waveguide ridge device. The individual substacks are indicated by colored letters with a decreasing emission wavelength from A to E. The insets show the band structure calculations of regions A and E. Two optical transitions occur between the upper lasing level |3⟩ and the lower lasing levels |2⟩ and |2′⟩, which also act as the extractor state. Level |1⟩ is the injector state for the next period. The lower lasing levels |2⟩ and |2′⟩ are depopulated to level |1⟩ by the emission of a LO-phonon.

The substacks in the stacked heterostructure are arranged in an ABCDE structure. Figure 1 shows a sketch of a ridge device. The colored letters on the facet indicate the location of the five stacks with decreasing emission wavelength from A to E. Band structure calculations of regions A and E are displayed in the insets. Although the emission wavelength of stacks A and E are significantly different; the shape of the band structure is rather similar, resulting in a stable performance over the whole substack range. Each stack exhibits two optical transitions from level |3⟩ to level |2⟩ and |2′⟩, respectively, where level |2′⟩ also acts as the extractor state. Electrons are depopulated by the emission of a LO-phonon to level |1⟩, which injects the electrons into the next period.

## MBE Growth and Device Fabrication

The samples were grown in a Riber Compact 21 molecular beam epitaxy (MBE) system on free-standing n+ GaAs (100) substrates. To ensure the high crystal quality of the structures, the THz QCL was measured with triple-axis high-resolution X-ray diffraction (HRXRD). To realize the structure as discussed in the design section above required multiple iterations. We started initially with an ABCDEDCBA structure. The processed device showed multiple kinks in its LIV curve. The designed lasing threshold was shifted by each stacked active region and needed to be adapted to inhibit absorption. In order to do this, regions A, C, and E were grown as single-stack active regions. The separate growth of the single-stack THz QCL structures allowed us to extract the required informations about the current densities at the maximum signal and emission wavelengths. Based on the light–current–voltage (LIV) measurement results of these single stacks, the design and the doping of each substack design was adjusted to obtain the desired wavelengths at the same current densities. The LIV measurements of the single-stacks A, C and E can be found in Supporting Information. Building up on this information we adjusted the doping for the substacks B and D. The doping was adjusted in a way, that the current densities at the maximum signal are matched for each substack. Subsequently, an ABCDE structure with the modified parameters obtained from the single-stack measurements was grown. After a further iteration, an ABCDE structure (C1107_ElMonstro) was grown that contains slightly adjusted stacks B and D, to optimize their emission wavelength. The number of periods per substack and their centered emission frequencies are shown in Table 1. The number of periods is adjusted according to the output power obtained by the single-stack measurements to normalize the gain for all five designs. For the detailed growth sheet, see Supporting Information. The thickness of the final ABCDE active region is 13 μm.

**Table 1:** Number of Periods and Centered Emission Frequencies of the Five Different Sub-Stacks^a^

region	periods	emission frequency
A	52	4.4 THz, 3.9 THz
B	56	4.2 THz, 3.8 THz
C	58	3.8 THz, 3.3 THz
D	62	3.4 THz, 2.7 THz
E	66	3.3 THz, 2.4 THz

a The number of periods was adjusted to normalize the gain.

The grown structure is processed into double-metal waveguides (DMWGs), providing high optical confinement and low optical losses.^18^ Ridge resonators with different dimensions ranging from 60 to 120 μm in width and 1 to 3.3 mm in length were fabricated. The height of the resonators is 13 μm. A standard process for DMWGs was used for the fabrication. First, the active region sample is flip-chip bonded onto a n+ GaAs chip by Au–Au thermocompression wafer bonding. Afterward, the active region substrate is removed by polishing and chemical wet etching. The final ridge resonators are defined by means of optical lithography and dry etching. For the top and bottom contact, Ti/Au metallization (5/500 nm and 5/1000 nm, respectively) is used. The laser chip is indium soldered to a copper plate, and bond wires are used for electrical contacting. The copper plate is then mounted onto a coldfinger of a He flow cryostat for the measurement.

## Experimental Results

The devices are driven by a voltage pulse generator (AVTECH AVR-3HF-B) in pulsed operation or by a DC source-measurement-unit (Keithley 2602A) in CW operation. The emitted light is guided into a Fourier-transform infrared spectrometer (Bruker Vertex 80, Mylar multilayer beam splitter T222/IR) by a parabolic mirror, which records spectra with a resolution of 2.25 GHz. The light intensity in pulsed operation is detected by the internal DTGS detector of the FTIR. For the power measurements in cw mode, a calibrated thermopile detector (Dexter 6M), which is integrated in the cryostat housing, is used.

**Figure 2:**
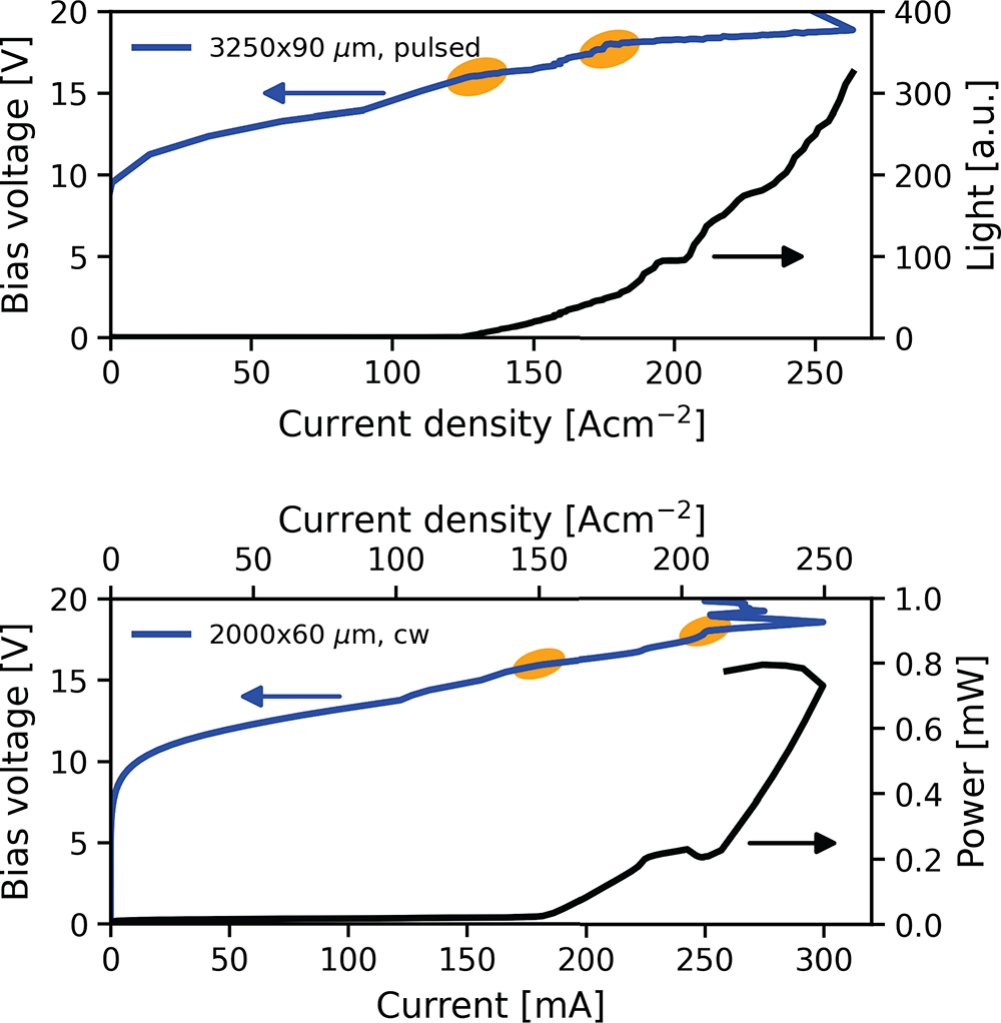
Comparison of the light–current–voltage (LIV) curves of two devices in pulsed and CW operation. Top: LIV curve of a 3.25 mm long and 90 μm wide ridge device in pulsed operation with a repetition rate of 100 kHz, a pulse length of 500 ns and a modulation frequency of 10 Hz. Bottom: LIV curve of 2 mm long and 60 μm wide ridge device in continuous-wave operation. Both measurements were performed at a heat sink temperature of 5 K. The kinks highlighted in both plots indicate the onset of photon-driven transport.

The electrical and optical performance of two different devices is depicted in Figure 2. On top, the LIV of a 3.25 mm long and 90 μm wide ridge in pulsed operation with a repetition rate of 100 kHz, a pulse length of 500 ns, and a modulation frequency of 10 Hz is shown. The maximum operating temperature in pulsed operation is 143 K. The bottom plot shows the LIV curve of a 2 mm long and 60 μm wide ridge in continuous-wave (CW) operation. Both measurements are conducted at a heat sink temperature of 5 K. As illustrated in the bottom plot, the device exhibits a threshold current density of 150 A cm^–2^ and single-facet output powers up to 800 μW. The optical output power of the device is measured by using a thermopile detector. In CW operation, the maximum operating temperature is 58.5 K. All devices, regardless of their dimensions, work in CW operation. In both operation modes, pulsed and CW, the IVs exhibit characteristic kinks, where the current is increasing (highlighted with orange circles in Figure 2). The faster increasing current after the kink is referred to as the onset of photon-assistant transport.^19^ In this active region, more than one lasing kink can be observed due to the staggered start of the emission from the different regions A–E.

**Figure 3:**
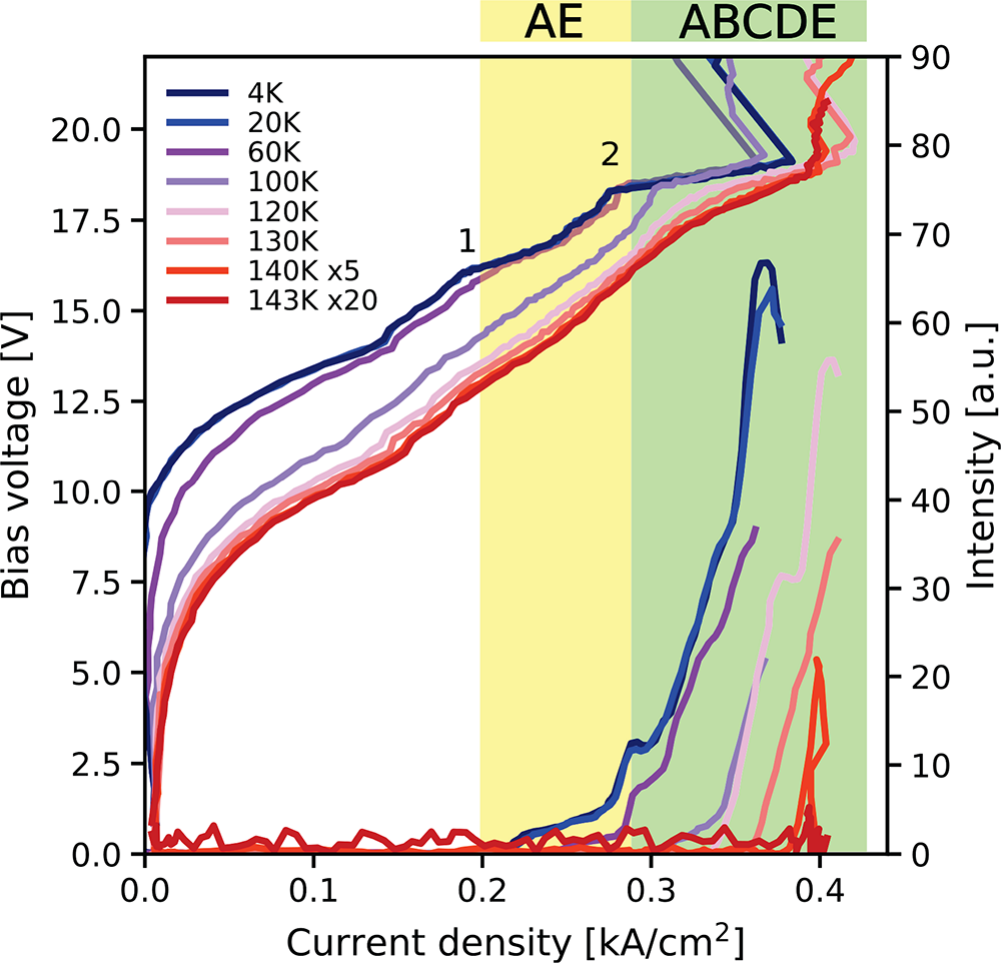
Temperature-dependent light–current–voltage curve of a 3.25 mm long and 60 μm wide ridge device at different heat sink temperatures in a pulsed operation with a repetition rate of 20 kHz and a pulse width of 500 ns. Bias points 1 and 2 on the current–voltage curve indicate where different regions start to lase. At bias point 1, which corresponds to the lasing threshold, regions A and E begin to lase. After bias point 2, all five regions A–E contribute to the lasing operation. The device works up to a maximum operating temperature of 143 K.

In order to investigate the behavior of the active region at elevated temperatures, we performed temperature-dependent LIV measurements. Figure 3 shows the electrical and optical performances of a 3.25 mm long and 60 μm wide ridge laser at different heat sink temperatures. The device was driven in pulsed operation with a repetition rate of 20 kHz and a pulse width of 500 ns (1% duty cycle) to prevent heating of the structure during laser operation. For low temperatures, two lasing kinks are clearly measured on the IV curve. At bias point 1, the device starts to lase at photon energies emitted from regions A and E. After bias point 2, the output power increases significantly, which corresponds to the emission of all five regions A–E. The different active regions are designed in a way that the currents at the maximum output power are matched. Due to the different dynamic ranges of the substacks, the threshold currents are shifted, resulting in the two lasing kinks in the IV curve. With the rising heat sink temperature, the threshold current density of the individual substacks shifts to higher values because of increased thermally induced leakage channels. The maximum operating temperature in the pulsed operation is 143 K, which is a record value for a heterogeneous THz QCL.

**Figure 4:**
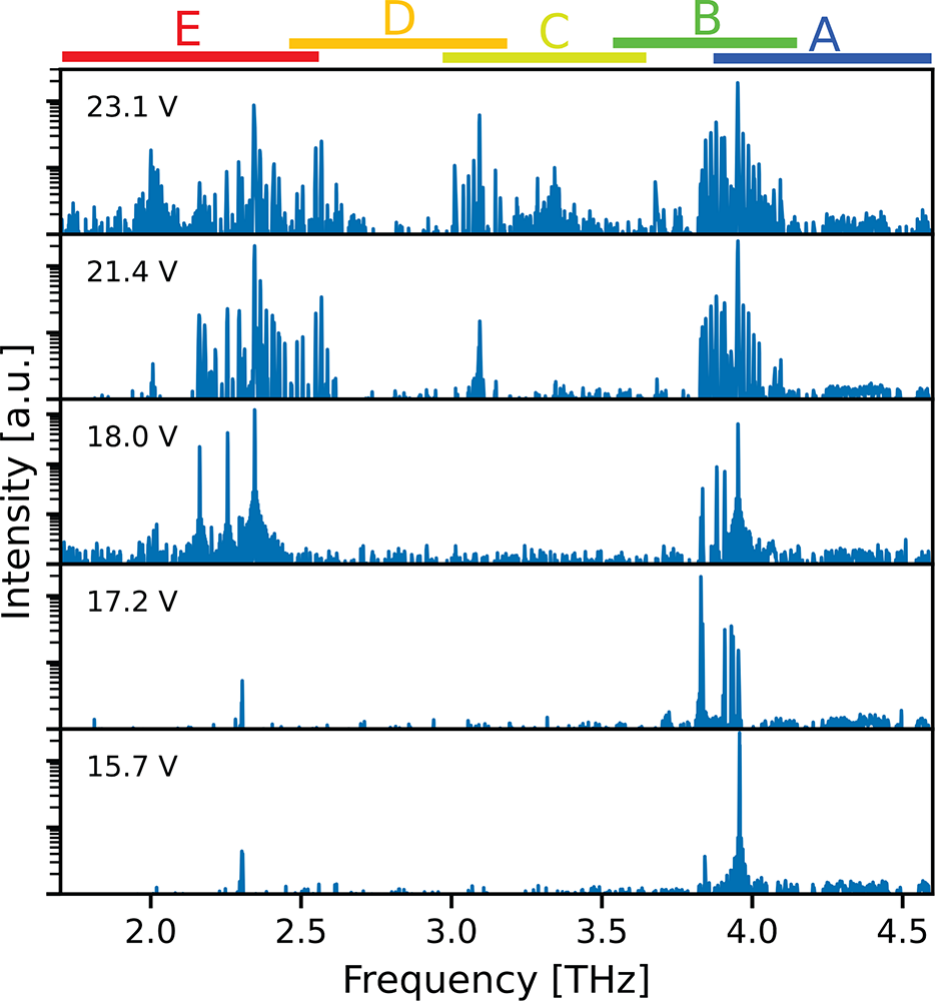
Mode evolution in pulsed operation for a 2 mm long and 60 μm wide ridge device at a heat sink temperature of 5 K. Above threshold, modes at the edges of the gain region start to lase. By raising the bias voltage/current, the spectrum broadens and the emission covers the whole gain region between 1.9 and 4.5 THz. The *y*-axis is shown in log-scale. The colored bars on top indicate the frequency range of each substack, respectively.

Figure 4 shows the spectral mode evolution of a 2 mm long and 60 μm wide ridge device in a pulsed operation (100 kHz, 500 ns, 5 K). The first modes appear on the edges of the gain spectrum. With the rising bias voltage, the mode profile broadens, and the gap between the two starting regions is continuously filled. The maximum emission spectrum ranges from 1.9 to 4.5 THz, spanning more than one octave. Additionally, measurements using high-pass and low-pass filters (cutoff frequency 3 GHz, respectively) are performed in order to prove that the modes do not originate from electronic mixing processes on the detector (see Supporting Information).

**Figure 5:**
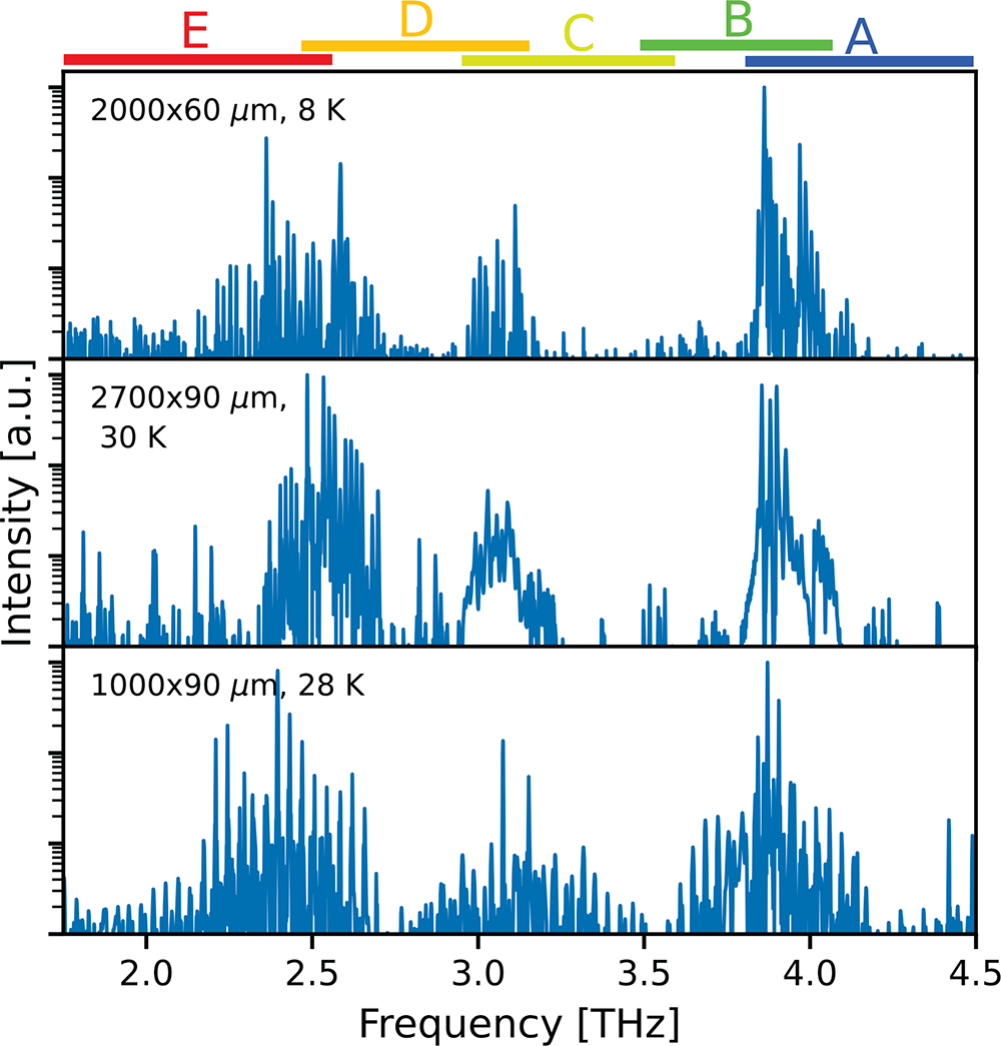
Broadband spectra of three differently sized devices in CW operation. They show emission over the whole gain region in CW operation. The spectra of the ridge devices, 2000 × 60 μm, 2700 × 90 μm, and 1000 × 90 μm, are measured at a heat sink temperature of 28, 30, and 8 K, respectively. The colored bars on top indicate the frequency range of each substack, respectively. The *y*-axis is shown in log-scale.

In order to rule out averaging effects during the measurements in pulsed operation and to demonstrate, that all stacks lase at the same time, we perform measurements in CW operation. Figure 5 shows the broad emission spectra of three devices with different dimensions. The emission bandwidth of the heterogeneous THz QCL is remarkable if one considers the emission spectra of the single-stack active regions (see Supporting Information). The emission bandwidth of these single-stack active regions is comparatively narrow. In terahertz time-domain spectroscopy measurements from a different heterogeneous THz QCL^20^ it was shown, that the photon emission of the individual stacks strongly interacts with each other. Consequently, this leads to an optical seeding of yet nonlasing stacks by the already lasing regions. This fact helps the nonlasing stacks to reach the lasing threshold even though the inversion induced by electrical pumping would not be sufficient to compensate for the losses. Furthermore, due to the overlap of the gain spectra of the different substacks, low-gain regions are stimulated additionally, resulting in the observed broadband emission spectrum.

In conclusion, we demonstrated a heterogeneous THz QCL consisting of five different active regions, based on a three-well, LO-phonon depopulation design. Devices show broadband emission in pulsed and CW operation covering a frequency range of 2.6 THz between 1.9 to 4.5 THz. In CW operation, peak output powers of 800 μW are detected. The maximum operating temperature is 58.5 K in CW operation and 143 K in pulsed operation. The latter is a record value for heterogeneous THz QCLs. Compared to other heterogeneous THz QCLs, which were based on bound-to-continuum or quasi-bound-to-continuum designs, the structure reported in this work successfully combines the high-temperature operation of the optimized three-well design^16^ with ultrabroadband emission.

## Supporting Information Available

Growth sheet of the final active region, single-stack active region measurements, and filter measurements (PDF)
