# Supplementary material for: Ultrabroadband Heterogeneous THz Quantum Cascade Laser
Source: ACS Photonics. 2022 Dec 21;10(1):111–5. doi: 10.1021/acsphotonics.2c01202 (PMC9853851; doi:10.1021/acsphotonics.2c01202)
Supplement: Supplementary file 1 — ph2c01202_si_001.pdf [file ph2c01202_si_001.pdf]

Supporting Information for

Ultra-Broadband Heterogeneous THz Quantum Cascade Laser

Michael Jaidl[1,2,*], Maximilian Beiser[2,3,*], Miriam Giparakis[2,3], Martin Alexander Kainz[1,2], Dominik Theiner[1,2], Benedikt Limbacher[1,2], Marie Christine Ertl[1,2], Aaron Maxwell Andrews[2,3], Gottfried Strasser[2,3], Juraj Darmo[1,2], Karl Unterrainer[1,2]

[1]Photonics Institute, TU Wien, Gusshausstrasse 27-29, 1040 Vienna, Austria
[2]Center for Micro- and Nanostructures, TU Wien, Gusshausstrasse 25a, 1040 Vienna, Austria
[3]Institute of Solid State Electronics, TU Wien, Gusshausstrasse 25a, 1040 Vienna, Austria

michael.jaidl@tuwien.ac.at; maximilian.beiser@tuwien.ac.at

This Supporting Information document includes:

Table S1: Growth sheet of the final heterogeneous THz QCL consisting of five different active region designs.

Figure S1: Light-current-voltage curves of the single-stack active regions A, C and E used for the doping and emission wavelength adjustment.

Figure S2: Spectra of the single-stack active regions A, C and E.

Figure S3: Spectral measurements using high-pass and low-pass filters.

**Layer (Substrate → Surface)**	**X (%)**	**Thickness (nm)**	**Doping (Si)**
n+ GaAs Substrate		650±20 µm	
GaAs		300	
AlGaAs	55	150	
Si: GaAs		70	5 e18
Loop 52			
AlGaAs	*24*	*4.6*	
GaAs		*8.95*	
AlGaAs	*24*	*2.03*	
GaAs		*9*	
AlGaAs	*24*	*4*	
GaAs		*4*	
GaAs		*4.12*	*3e16*
GaAs		*8.58*	
Endloop			
Loop 56			
AlGaAs	*24*	*4.6*	
GaAs		*9.02*	
AlGaAs	*24*	*2.07*	
GaAs		*8.84*	
AlGaAs	*24*	*4.02*	
GaAs		*4*	
GaAs		*4.11*	*3e16*
GaAs		*8.55*	
Endloop			
Loop 58			
AlGaAs	*24*	*4.6*	

GaAs		*9.2*	
AlGaAs	*24*	*2.25*	
GaAs		*8.5*	
AlGaAs	*24*	*4.12*	
GaAs		*4*	
GaAs		*4.02*	*3e16*
GaAs		*8.48*	
Endloop			
Loop 62			
AlGaAs	*24*	*4.6*	
GaAs		*9.38*	
AlGaAs	*24*	*2.36*	
GaAs		*8.21*	
AlGaAs	*24*	*4.22*	
GaAs		*4*	
GaAs		*3.44*	*3e16*
GaAs		*8.95*	
Endloop			
Loop 66			
AlGaAs	*24*	*4.6*	
GaAs		*9.5*	
AlGaAs	*24*	*2.4*	
GaAs		*8*	
AlGaAs	*24*	*4.28*	
GaAs		*4*	
GaAs		*2.86*	*3e16*
GaAs		*9.43*	
Endloop			

Table S1: Growth sheet of the final heterogeneous THz QCL consisting of 5 different active region designs.

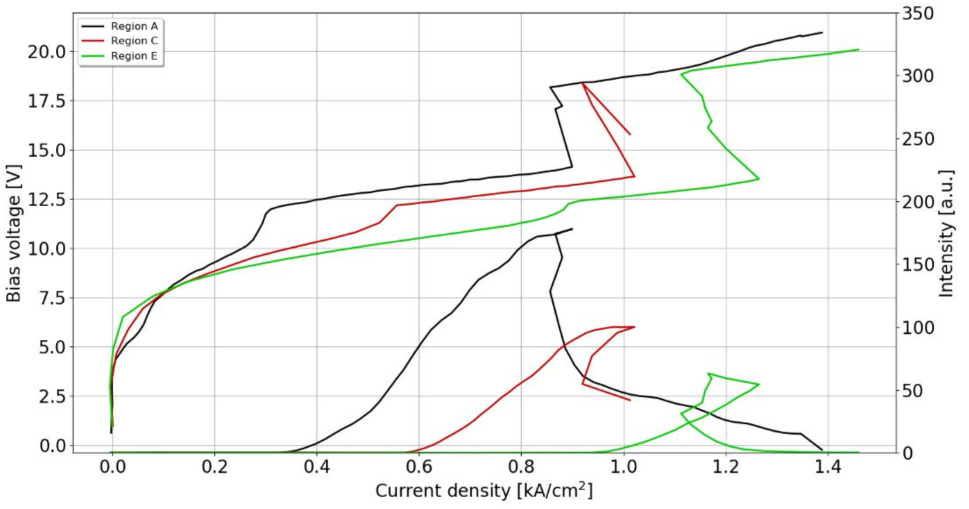

Figure S1: Light-current-voltage curves of the single-stack active regions A, C and E used for the doping and emission wavelength adjustment.

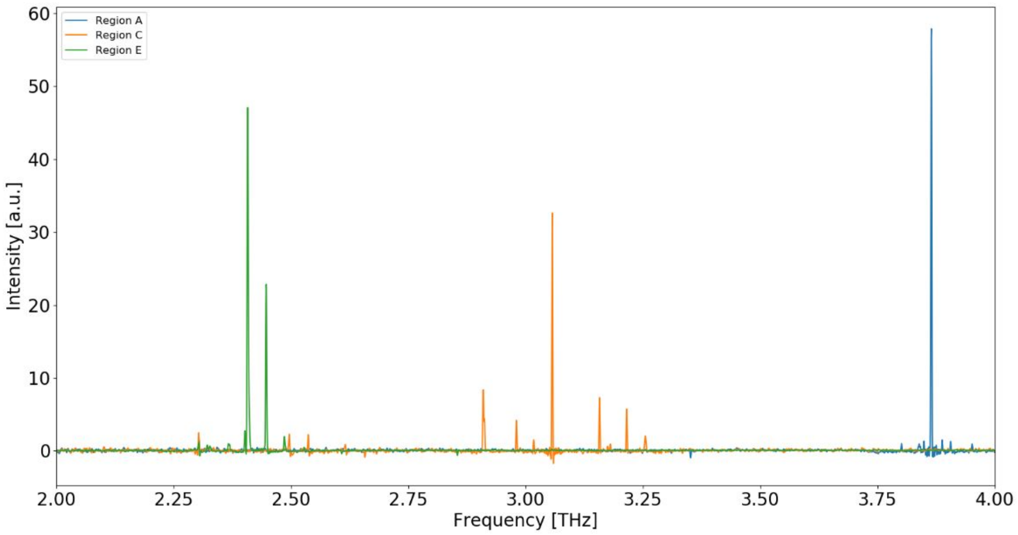

Figure S2: Spectra of the single-stack active regions A, C and E

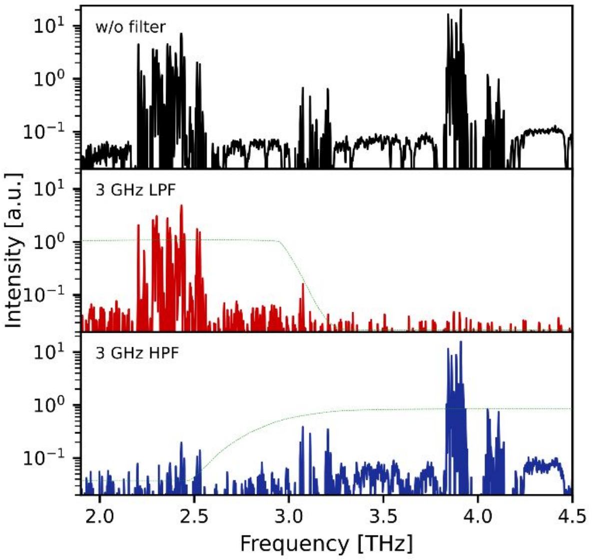

Figure S3: Spectral measurements of a 90-µm-wide and 2.7-mm-long ridge laser using high-pass and low-pass filters (cut-off frequency 3 GHz, respectively). The green lines correspond to the filter responses.
